# Synthesis and crystal structure of 2-chloro-1-(3-hy­droxy­phen­yl)ethanone

**DOI:** 10.1107/S2056989022009835

**Published:** 2022-10-20

**Authors:** Prabhakar Priyanka, Bidarur K. Jayanna, Vinaya, Holehundi J. Shankara Prasad, Thayamma R. Divakara, Hemmige S. Yathirajan, Sean Parkin

**Affiliations:** a Department of Chemistry, B. N. M. Institute of Technology, Bengaluru-560 070, India; b Department of Studies in Chemistry, University of Mysore, Manasagangotri, Mysuru-570 006, India; c Department of Chemistry, Yuvaraja’s College, University of Mysore, Mysore-570 005, India; d T. John Institute of Technology, Bengaluru-560 083, India; e Department of Chemistry, University of Kentucky, Lexington, KY, 40506-0055, USA; Katholieke Universiteit Leuven, Belgium

**Keywords:** crystal structure, α-haloketone, chlorinated aceto­phenone

## Abstract

The structure of 2-chloro-1-(3-hy­droxy­phen­yl)ethanone, C_8_H_7_ClO_2_, an α-halo­ketone is described. The mol­ecule is planar (r.m.s. deviation = 0.0164 Å) and in the crystal, inversion-symmetric dimers are formed as a result of pairs of strong O—H⋯O and weak C—H⋯O hydrogen bonds. A brief comparison is made with structurally related compounds deposited in the CSD. In addition, the synthesis and some spectroscopic details are presented.

## Abstract 2

The synthesis, crystal structure, and some spectroscopic details for 2-chloro-1-(3-hy­droxy­phen­yl) ethanone, C_8_H_7_O_2_Cl, an α-haloketone of use in organic synthesis, are described.

## 1. Chemical context

α-Haloketones have proven to be useful building blocks for the preparation of compounds of various classes because of their high reactivity and selective transformations with a variety of reagents (Erian *et al.*, 2003 ▸). Chlorinated aceto­phenones are widely used in organic synthesis as inter­mediates for the manufacture of active pharmaceutical ingredients (Ott-Dombrowski *et al.*, 2019 ▸). For example, 2-chloro-1-(4-hy­droxy­phen­yl)ethanone is a reagent that is used in the preparation of hy­droxy­pyrimidine derivatives for their HDAC (histone de­acetyl­ase) inhibitory activity (Kemp *et al.*, 2011 ▸). In light of the importance of α-haloketones, this paper reports the synthesis, crystal structure, and some spectroscopic details for the title compound, C_8_H_7_O_2_Cl, (**I**). 
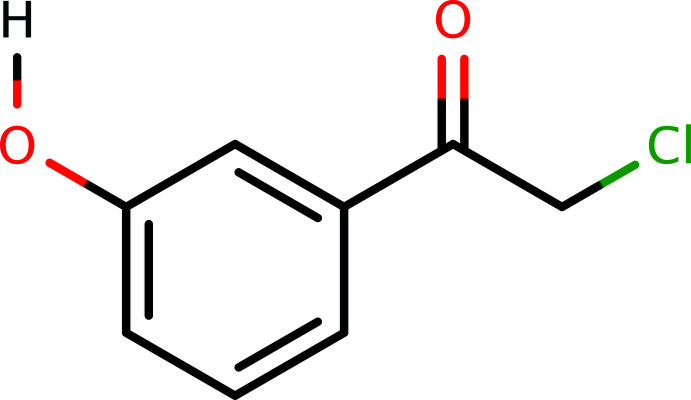


## 2. Structural commentary

**Figure 1:**
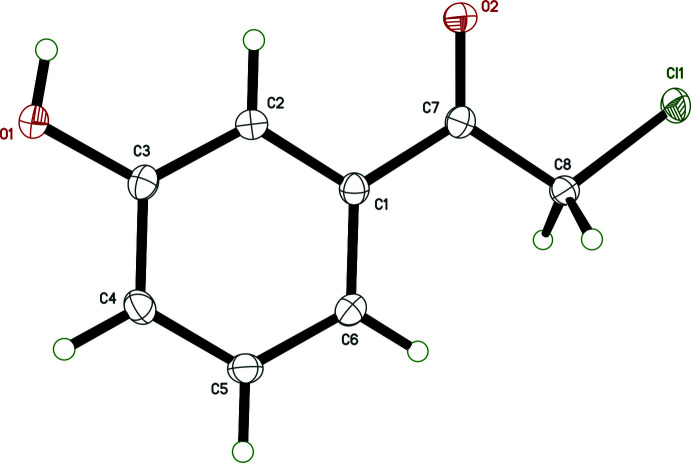
An ellipsoid plot (50% probability) of **I**. Hydrogen atoms are drawn as small circles.

The mol­ecule of **I** (Fig. 1 ▸) is planar (r.m.s. deviation = 0.0164 Å), with the largest deviation being for Cl1, which is 0.0346 (5) Å from the mean plane through all non-H atoms due to the O2—C7—C8—Cl1 torsion angle of −2.07 (14)°. The hydroxyl hydrogen atom, H1*O*, which was refined freely, lies 0.045 (16) Å out of the mean plane, with a C2—C3—O1—H1*O* torsion angle of 1.8 (12)°, its position being mandated by inter­molecular hydrogen bonding (see section 3, *Supra­molecular details*). All bond lengths and angles fall within the expected ranges for organic structures.

## 3. Supra­molecular features

**Figure 2:**
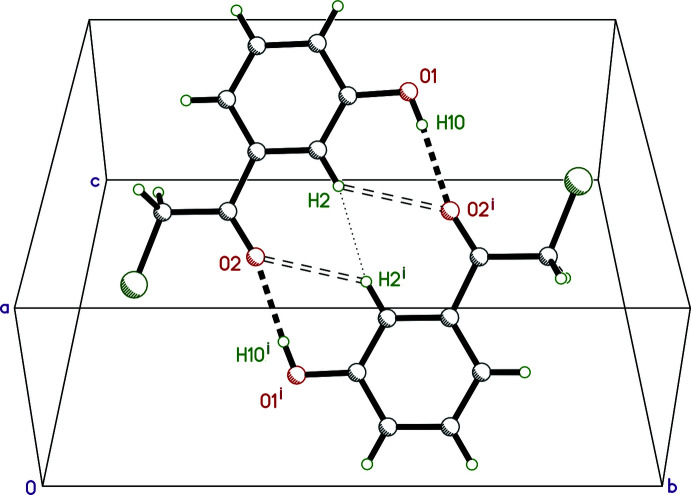
A partial packing plot showing the main supra­molecular motif in **I**: a hydrogen-bonded dimer between inversion-related [symmetry code: (i) −*x* + 1, −*y* + 1, −*z* + 1] mol­ecules. Strong O—H⋯O hydrogen bonds are shown as thick dashed lines, weaker C—H⋯O inter­actions as open dashed lines, and an unfavourable, forced close contact between hydrogen atoms as a dotted line.

**Figure 3:**
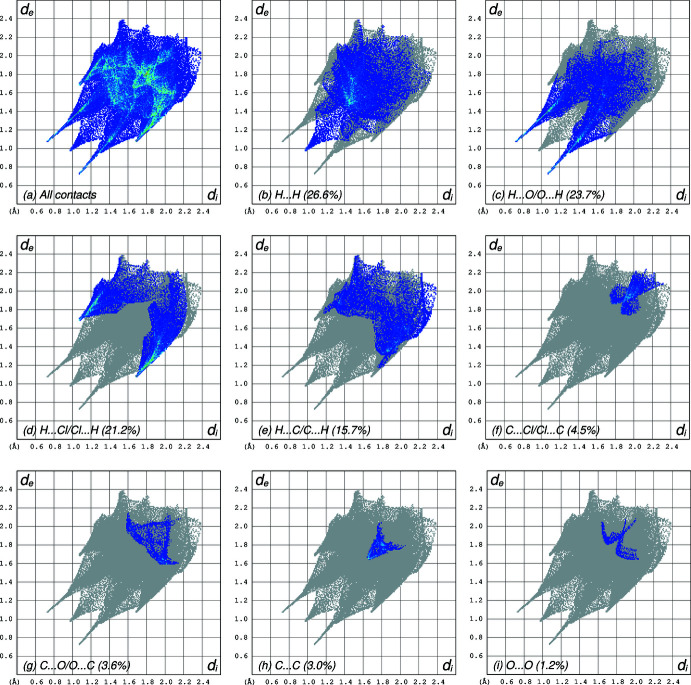
Hirshfeld surface fingerprint plots showing the relative contributions of various atom–atom contacts in the packing of **I**. (*a*) All contacts, (*b*) H⋯H (26.6%), (*c*) H⋯O/O⋯H (23.7%), (*d*) H⋯Cl/Cl⋯H (21.2%), (*e*) H⋯C/C⋯H (15.7%), (*f*) C⋯Cl/Cl⋯C (4.5%), (*g*) C⋯O/O⋯C (3.6%), (*h*) C⋯C (3.0%), (i) O⋯O (1.2%). All other contact types are <1%.

The main supra­molecular feature is an inversion dimer resulting from a pair of symmetrically equivalent hydrogen bonds, O1—H1*O*⋯O2^i^ and O1^i^—H1*O* ^i^⋯O2 [symmetry code: (i) −*x* + 1, −*y* + 1, −*z* + 1], giving an 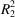 (14) motif. The cohesion of this dimer is augmented by a pair of weak hydrogen bonds, C2—H2⋯O2^i^ and C2^i^—H2^i^⋯O2 (Table 1 ▸). It also, however, brings inversion-related H2 atoms into unfavourably close proximity [H2⋯H2^i^ = 2.22 (3) Å]. These inter­actions are all illustrated in Fig. 2 ▸. Other noteworthy inter­molecular contacts are weak C8—H8⋯O1^ii^ [symmetry code: (ii) −*x* + 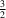 , *y* − 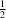 , −*z* + 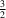 ] inter­actions between 2_1_ screw-related mol­ecules, which loosely connect the dimers into layers parallel to (10 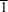 ). Almost all of the atom–atom contact coverages qu­anti­fied in a Hirshfeld-surface analysis using *CrystalExplorer* (Spackman *et al.*, 2021 ▸) involve hydrogen (H⋯H = 26.6%, H⋯O/O⋯H = 23.7%, H⋯Cl/Cl⋯H = 21.2%, H⋯C/C⋯H = 15.8%), with all other contact types being <5%. Further details are given in individual Hirshfeld-surface fingerprint plots (Fig. 3 ▸).

## 4. Database survey

A search of the Cambridge Structure Database (v5.43 with updates as of June 2022; Groom *et al.*, 2016 ▸) for a search fragment consisting of the structure of **I** but with the OH and Cl groups replaced by ‘any non-H’ gave 71 hits. If the Cl site is specified as ‘any halogen’, there are just four hits, only three of which are unique, and all have Br as the halogen. Structure AWOCAS (Aldeborgh *et al.*, 2014 ▸) is chemically a Br analogue of **I**, but its crystal structure is quite different (triclinic *P* 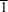 *vs P*2_1_/*n* for **I**). QAJNAS (Jasinski *et al.*, 2011 ▸) [and QAJNAS01 (Mounir *et al.*, 2013 ▸)] has NO_2_ in place of the hydroxyl. Lastly, MEXCOJ (Ambekar *et al.*, 2013 ▸) has OC=OPh in place of the OH in **I**. Other similar structures in the literature include: LEFNAN (Fun *et al.*, 2012 ▸), which is the 4-hy­droxy­phenyl analogue of **I** and crystallizes with the symmetry of *P*2_1_/*c*; FUHHOG (Qing & Zhang, 2009 ▸), which is the bromo analogue of LEFNAN; and CUYDOR (Mei *et al.*, 2015 ▸), which has 4-fluoro­phenyl in place of the halogen of LEFNAN and FUHHOG.

## 5. Synthesis, crystallization and spectroscopic details

**Figure 4:**
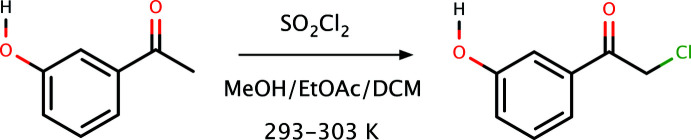
The overall reaction scheme for the synthesis of **I**.

*Synthesis and crystallization*: For the synthesis of **I**, sulfuryl chloride (150 mg, 1.1 mmol) was added dropwise to a stirred mixture of 3-hy­droxy­aceto­phenone (100 mg, 0.74 mmol) in 5 ml of methanol and 10 ml of ethyl acetate/di­chloro­methane at 293–303 K. After completion of the addition, it was allowed to return to RT with stirring for 1 h. The reaction was monitored by TLC. Then the solvent was removed under reduced pressure by rotary evaporation to give the desired product in 95% yield. An overall reaction scheme is depicted in Fig. 4 ▸. X-ray quality crystals were obtained by crystallization from ethanol (m.p. 352–354 K).

*Spectroscopic data*: Infrared and NMR spectroscopic details are as follows.

FTIR (γ in cm^−1^): 3400 (Ar—OH, broad), 2987 (C—H stretching), 1694 (C=C stretching), 1789 (*s*, C=O stretching), 832 (*s*, Ar-C—H bending).

^1^H NMR: CDCl_3_ (400 MHz, δ ppm): 4.7 (*s*, 2H, –CH_2_), 5.671 (*s*, 1H, –OH), 7.14 (*d*, 1H, Ar—H, *J* = 4.8 Hz), 7.36–7.4 (*t*, 2H, Ar—H, *J* = 16 Hz), 7.493–7.51 (*m*, 1H, Ar—H, *J* = 6.4 Hz).

## 6. Refinement

Crystal data, data collection, and structure refinement details are given in Table 2 ▸. All hydrogen atoms were found in difference-Fourier maps, but subsequently, the carbon-bound hydrogens were included using riding models, with constrained distances set to 0.95 Å (C*sp* ^2^—H) and 0.99 Å (*R*_2_CH_2_). The hydroxyl hydrogen atom coordinates were refined freely. In all cases, *U*_iso_(H) values were set to 1.2*U*_eq_ of the attached atom.

## Supplementary Material

Crystal structure: contains datablock(s) I, global. DOI: 10.1107/S2056989022009835/vm2272sup1.cif

Structure factors: contains datablock(s) I. DOI: 10.1107/S2056989022009835/vm2272Isup2.hkl

Supporting information file. DOI: 10.1107/S2056989022009835/vm2272Isup3.cml

CCDC reference: 2211527

Additional supporting information:  crystallographic information; 3D view; checkCIF report

## Figures and Tables

**Table 1:** Hydrogen-bond geometry (Å, °)

*D*—H⋯*A*	*D*—H	H⋯*A*	*D*⋯*A*	*D*—H⋯*A*
O1—H1*O*⋯O2^i^	0.803 (17)	2.004 (18)	2.8029 (12)	173.4 (16)
C2—H2⋯O2^i^	0.945 (15)	2.547 (15)	3.2633 (14)	132.7 (11)
C8—H8*A*⋯O1^ii^	0.99	2.36	3.3485 (14)	176

Symmetry codes: (i) 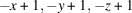 ; (ii) 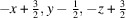 .

**Table 2:** Experimental details

Crystal data	
Chemical formula	C_8_H_7_ClO_2_
*M*_r_	170.59
Crystal system, space group	Monoclinic, *P*2_1_/*n*
Temperature (K)	90
*a*, *b*, *c* (Å)	4.9172 (2), 12.7016 (4), 11.8573 (3)
β (°)	96.294 (1)
*V* (Å^3^)	736.10 (4)
*Z*	4
Radiation type	Mo *K*α
μ (mm^−1^)	0.46
Crystal size (mm)	0.25 × 0.22 × 0.19

Data collection	
Diffractometer	Bruker D8 Venture dual source
Absorption correction	Multi-scan (*SADABS*; Krause *et al.*, 2015 ▸)
*T*_min_, *T*_max_	0.855, 0.971
No. of measured, independent and observed [*I* > 2σ(*I*)] reflections	12264, 1685, 1569
*R*_int_	0.028
(sin θ/λ)_max_ (Å^−1^)	0.650

Refinement	
*R*[*F* ^2^ > 2σ(*F* ^2^)], *wR*(*F* ^2^), *S*	0.024, 0.067, 1.04
No. of reflections	1685
No. of parameters	106
H-atom treatment	H atoms treated by a mixture of independent and constrained refinement
Δρ_max_, Δρ_min_ (e Å^−3^)	0.35, −0.18

Computer programs: *APEX3* (Bruker, 2016 ▸), *SHELXT* (Sheldrick, 2015*a* ▸), *SHELXL2019/2* (Sheldrick, 2015*b* ▸), *XP* in *SHELXTL* and *SHELX* (Sheldrick, 2008 ▸) and *publCIF* (Westrip, 2010 ▸).

## supplementary crystallographic information

### Crystal data

**Table:**

C_8_H_7_ClO_2_	*F*(000) = 352
*M**_r_* = 170.59	*D*_x_ = 1.539 Mg m^−^^3^
Monoclinic, *P*2_1_/*n*	Mo *K*α radiation, λ = 0.71073 Å
*a* = 4.9172 (2) Å	Cell parameters from 9994 reflections
*b* = 12.7016 (4) Å	θ = 2.4–27.5°
*c* = 11.8573 (3) Å	µ = 0.46 mm^−^^1^
β = 96.294 (1)°	*T* = 90 K
*V* = 736.10 (4) Å^3^	Cut block, colourless
*Z* = 4	0.25 × 0.22 × 0.19 mm

### Data collection

**Table:**

Bruker D8 Venture dual source diffractometer	1685 independent reflections
Radiation source: microsource	1569 reflections with *I* > 2σ(*I*)
Detector resolution: 7.41 pixels mm^-1^	*R*_int_ = 0.028
φ and ω scans	θ_max_ = 27.5°, θ_min_ = 2.4°
Absorption correction: multi-scan (*SADABS*; Krause *et al.*, 2015)	*h* = −6→6
*T*_min_ = 0.855, *T*_max_ = 0.971	*k* = −15→16
12264 measured reflections	*l* = −15→15

### Refinement

**Table:**

Refinement on *F*^2^	Primary atom site location: structure-invariant direct methods
Least-squares matrix: full	Secondary atom site location: difference Fourier map
*R*[*F*^2^ > 2σ(*F*^2^)] = 0.024	Hydrogen site location: mixed
*wR*(*F*^2^) = 0.067	H atoms treated by a mixture of independent and constrained refinement
*S* = 1.04	*w* = 1/[σ^2^(*F*_o_^2^) + (0.0323*P*)^2^ + 0.366*P*] where *P* = (*F*_o_^2^ + 2*F*_c_^2^)/3
1685 reflections	(Δ/σ)_max_ < 0.001
106 parameters	Δρ_max_ = 0.35 e Å^−^^3^
0 restraints	Δρ_min_ = −0.18 e Å^−^^3^

### Special details

**Table:**

Geometry. All esds (except the esd in the dihedral angle between two l.s. planes) are estimated using the full covariance matrix. The cell esds are taken into account individually in the estimation of esds in distances, angles and torsion angles; correlations between esds in cell parameters are only used when they are defined by crystal symmetry. An approximate (isotropic) treatment of cell esds is used for estimating esds involving l.s. planes.

### Fractional atomic coordinates and isotropic or equivalent isotropic displacement parameters (Å^2^)

**Table:**

	*x*	*y*	*z*	*U*_iso_*/*U*_eq_	
O1	1.05641 (18)	0.59830 (6)	0.66167 (7)	0.01786 (19)	
H1O	0.938 (3)	0.6217 (12)	0.6166 (14)	0.021*	
O2	0.38728 (17)	0.32908 (6)	0.48648 (7)	0.01810 (19)	
Cl1	0.22134 (5)	0.10971 (2)	0.48449 (2)	0.01719 (10)	
C1	0.7757 (2)	0.33060 (8)	0.62318 (9)	0.0131 (2)	
C2	0.8093 (2)	0.43878 (9)	0.60814 (9)	0.0140 (2)	
H2	0.686 (3)	0.4757 (12)	0.5553 (12)	0.017*	
C3	1.0190 (2)	0.49212 (9)	0.67174 (9)	0.0139 (2)	
C4	1.1997 (2)	0.43790 (9)	0.74969 (9)	0.0151 (2)	
H4	1.345037	0.474217	0.792591	0.018*	
C5	1.1662 (2)	0.33023 (9)	0.76434 (9)	0.0157 (2)	
H5	1.289511	0.293235	0.817524	0.019*	
C6	0.9546 (2)	0.27607 (9)	0.70220 (9)	0.0147 (2)	
H6	0.931888	0.202668	0.713351	0.018*	
C7	0.5426 (2)	0.27915 (8)	0.55286 (9)	0.0133 (2)	
C8	0.5108 (2)	0.16148 (9)	0.56861 (10)	0.0148 (2)	
H8A	0.493730	0.146717	0.649517	0.018*	
H8B	0.677307	0.125426	0.548476	0.018*	

### Atomic displacement parameters (Å^2^)

**Table:**

	*U*^11^	*U*^22^	*U*^33^	*U*^12^	*U*^13^	*U*^23^
O1	0.0212 (4)	0.0116 (4)	0.0191 (4)	−0.0027 (3)	−0.0048 (3)	0.0006 (3)
O2	0.0185 (4)	0.0146 (4)	0.0198 (4)	−0.0007 (3)	−0.0042 (3)	0.0025 (3)
Cl1	0.01466 (15)	0.01477 (15)	0.02144 (16)	−0.00314 (9)	−0.00114 (11)	−0.00204 (10)
C1	0.0128 (5)	0.0128 (5)	0.0137 (5)	−0.0003 (4)	0.0021 (4)	−0.0010 (4)
C2	0.0143 (5)	0.0132 (5)	0.0141 (5)	0.0006 (4)	−0.0001 (4)	0.0008 (4)
C3	0.0160 (5)	0.0120 (5)	0.0141 (5)	−0.0006 (4)	0.0030 (4)	−0.0012 (4)
C4	0.0142 (5)	0.0162 (5)	0.0146 (5)	−0.0004 (4)	−0.0004 (4)	−0.0032 (4)
C5	0.0159 (5)	0.0157 (5)	0.0150 (5)	0.0033 (4)	−0.0006 (4)	−0.0001 (4)
C6	0.0161 (5)	0.0121 (5)	0.0159 (5)	0.0010 (4)	0.0020 (4)	0.0000 (4)
C7	0.0136 (5)	0.0129 (5)	0.0138 (5)	0.0000 (4)	0.0029 (4)	−0.0006 (4)
C8	0.0125 (5)	0.0126 (5)	0.0184 (5)	−0.0011 (4)	−0.0020 (4)	0.0005 (4)

### Geometric parameters (Å, º)

**Table:**

O1—C3	1.3682 (13)	C3—C4	1.3922 (16)
O1—H1O	0.803 (17)	C4—C5	1.3906 (16)
O2—C7	1.2138 (14)	C4—H4	0.9500
Cl1—C8	1.7725 (11)	C5—C6	1.3892 (16)
C1—C6	1.3967 (15)	C5—H5	0.9500
C1—C2	1.3978 (15)	C6—H6	0.9500
C1—C7	1.4921 (15)	C7—C8	1.5164 (15)
C2—C3	1.3857 (15)	C8—H8A	0.9900
C2—H2	0.945 (15)	C8—H8B	0.9900
			
C3—O1—H1O	109.2 (11)	C6—C5—H5	119.6
C6—C1—C2	119.91 (10)	C4—C5—H5	119.6
C6—C1—C7	123.12 (10)	C5—C6—C1	119.35 (10)
C2—C1—C7	116.97 (10)	C5—C6—H6	120.3
C3—C2—C1	120.16 (10)	C1—C6—H6	120.3
C3—C2—H2	120.1 (9)	O2—C7—C1	121.59 (10)
C1—C2—H2	119.7 (9)	O2—C7—C8	121.91 (10)
O1—C3—C2	122.25 (10)	C1—C7—C8	116.50 (9)
O1—C3—C4	117.6 (1)	C7—C8—Cl1	112.57 (8)
C2—C3—C4	120.15 (10)	C7—C8—H8A	109.1
C5—C4—C3	119.57 (10)	Cl1—C8—H8A	109.1
C5—C4—H4	120.2	C7—C8—H8B	109.1
C3—C4—H4	120.2	Cl1—C8—H8B	109.1
C6—C5—C4	120.86 (10)	H8A—C8—H8B	107.8
			
C6—C1—C2—C3	−0.24 (16)	C2—C1—C6—C5	−0.61 (16)
C7—C1—C2—C3	179.06 (10)	C7—C1—C6—C5	−179.86 (10)
C1—C2—C3—O1	−178.62 (10)	C6—C1—C7—O2	178.50 (11)
C1—C2—C3—C4	0.97 (16)	C2—C1—C7—O2	−0.77 (15)
O1—C3—C4—C5	178.75 (10)	C6—C1—C7—C8	−1.61 (15)
C2—C3—C4—C5	−0.85 (16)	C2—C1—C7—C8	179.11 (9)
C3—C4—C5—C6	0.00 (17)	O2—C7—C8—Cl1	−2.07 (14)
C4—C5—C6—C1	0.73 (17)	C1—C7—C8—Cl1	178.05 (7)

### Hydrogen-bond geometry (Å, º)

**Table:**

*D*—H···*A*	*D*—H	H···*A*	*D*···*A*	*D*—H···*A*
O1—H1*O*···O2^i^	0.803 (17)	2.004 (18)	2.8029 (12)	173.4 (16)
C2—H2···O2^i^	0.945 (15)	2.547 (15)	3.2633 (14)	132.7 (11)
C8—H8*A*···O1^ii^	0.99	2.36	3.3485 (14)	176

Symmetry codes: (i) −*x*+1, −*y*+1, −*z*+1; (ii) −*x*+3/2, *y*−1/2, −*z*+3/2.
